# Evaluation of a new mouthwash formulated with Chlorhexidine and Cymenol after a scaling and root planing treatment in grade I and II periodontal patients

**DOI:** 10.4317/medoral.26818

**Published:** 2024-10-13

**Authors:** Paula Hermida-Cabrera, Felipe-Rodrigo Aguilera, Fernando Vivancos-Cuadras, Lourdes Ferrá-Domingo, Daniel Torres-Lagares, José-Luis Gutiérrez-Pérez, Tanya Pereira-Riveros, Teresa Vinuesa 2 María-Ángeles Serrera-Figallo, María Baus-Domínguez

**Affiliations:** 1 Department of Stomatology, Faculty of Dentistry, University of Seville, Seville, Spain; 2 Unit of Microbiology, Department of Pathology and Experimental Therapeutics, Faculty of Medicine and Health Sciences, University of Barcelona, L’Hospitalet de Llobregat, Barcelona, Spain; 3 School of Dentistry, Faculty of Medicine, Universidad Austral de Chile, Valdivia, Chile; 4 LACER Medical Department, Barcelona, Spain; 5 LACER R&D/Microbiology Department, Barcelona, Spain; 6 Hospital Universitario Virgen del Rocío, University of Seville, Seville, Spain

## Abstract

**Background:**

Periodontal disease is a multifactorial pathology whose treatment protocol is becoming increasingly standardized, relying, among others, on adjuvant therapies. This study aims to determine the efficacy of a new chlorhexidine compound compared with the gold standard 0.12% chlorhexidine during periodontal maintenance of a patient with periodontitis grade I or II after scaling and root planing treatment.

**Material and Methods:**

A parallel, randomized, double-blind clinical trial with two arms: (A) treatment with 0.12% chlorhexidine mouthwash; (B) treatment with new chlorhexidine mouthwash containing O-Cymen-5-Ol. The patient was examined: (D0) initial examination, (D7) clinical examination one week after treatment, (D14) clinical examination two weeks after treatment and (D28) clinical examination three weeks after treatment. They are collecting the following data: Plaque index (PI), gingival index (GI), bleeding index (BoP), pocket depth (PS), and attachment loss (CAL).

**Results:**

After a follow-up of 43 patients, no statistically significant differences were found between the groups at any of the times or for any of the parameters evaluated, meeting the predefined criteria of non-inferiority.

**Conclusions:**

The new chlorhexidine mouthwash proved to be non-inferior to 0.12% chlorhexidine in terms of efficacy during short-term periodontal maintenance after scaling and root planing.

** Key words:**Non-surgical periodontal therapy, plaque control, periodontitis, chlorhexidine, cymenol, mouthwash.

## Introduction

Periodontal disease is a pathology that has affected 48-68% of the world's population in the last ten years, according to recent systematic reviews and meta-analyses (1), showing a higher prevalence than studies conducted between the 1990s and 2010 (2).

This condition has a multifactorial etiology, with the periodontopathogenic flora (Porphyromonas *gingivalis*, Tannarella forsythia, and *Actinobacillus actinomycetemcomitans*) being one of its starting points (3).

Similarly, the accumulation of plaque and the individual's hygiene protocols accentuate its development in terms of severity and speed of implantation, as do those pathologies and deleterious habits with which it has been widely reported in the literature over the years (3,4).

Numerous articles speak of the relationship between certain systemic conditions such as diabetes mellitus or pregnancy with the increase in the prevalence of periodontal disease (5,6), as well as those studies that reflect the directly proportional and significant relationship between tobacco consumption and the development of the pathology, or more recently, with the consumption of vapers. However, this last association should be studied more extensively (3,4,7).

Periodontitis could be defined as a pathology induced by bacterial plaque that causes chronic inflammation and destroys the soft and hard tissues that support the teeth (8).

There have been numerous classifications of periodontal pathology over the years. The most current classification was presented in 2017 by the American Academy of Periodontology (AAP) together with the European Federation of Periodontology (EFP) (9). This classification classifies the pathology according to grades I, II, III, and IV (based on severity and complexity, extent and distribution) and stages A, B, and C (based on biological features that imply evidence of or risk of progression, anticipated response to treatment and systemic effects) (9).

Depending on the grade and stage of the patients, it is possible to determine the objectives and the consideration of the "success" of periodontal treatment. The primary phase of treatment consists of the active removal of subgingival and supragingival plaque deposits by scaling and root planing, followed by the maintenance phase (SPT), which aims to monitor the patient to ensure stability, the reduction of pockets and active disease to consider, if necessary, surgical treatment in those cases where non-surgical treatment has not achieved the desired objectives (8,10).

At the same time, the surgical and non-surgical approaches to the pathology are complemented by a variety of adjuvant treatments, ranging from antibiotics such as clarithromycin (based on the bacterial origin of the entity) (11) to photodynamic therapy (12,13) or the increasingly widespread use of hyaluronic acid (14).

Despite this, the adjuvant treatment par excellence, the most widely used in dental clinics, is 0.12% chlorhexidine mouthwash. There is "high scientific evidence" that this mouthwash reduces gingivitis in individuals with medium inflammation, prevents periodontitis, and reduces (15).

On the other hand, it is known that chlorhexidine, when used long-term, causes extrinsic staining of tooth enamel, which is why more and more product developers are looking for alternatives with similar success to this mouthwash (16).

Thus, this study aims to compare a new chlorhexidine compound containing cymenol (o-cymen-5-ol; C10H14O), an antimicrobial agent whose effect against bacteria and fungi has been briefly reported in the literature (17), with a 0.12% chlorhexidine compound during periodontal maintenance of patients with periodontitis grade I or II, after scaling and root planing treatment. For this, the null hypothesis (H0) defined implies that the 0.12% chlorhexidine + 0.10% cymenol formulation is not inferior to the 0.12% chlorhexidine formulation in terms of efficacy.

Materials and Methods

- Type of study

Parallel, randomized, double-masked, two-arm clinical trial approved by the Research Ethics Committee of the Rey Juan Carlos University (internal registration number: 0103202207122) and which complies with all the Guidelines of the Helsinki Declaration: Ethical Principles for Medical Research Involving Human Subjects, where the only invasive procedure performed on the patients was clinical examination through inspection and periodontal probing, after scaling and root planing treatment.

The present study was designed according to CONSORT guidelines for randomized controlled clinical trials.

The study groups were (A) treated with 0.12% chlorhexidine mouthwash.12% (B) treatment with new chlorhexidine mouthwash composed of Aqua, Glycerin, Pro-pylene Glycol, PEG-40 Hydrogenated Castor Oil, Xylitol, Sucralose, Chlorhexidine Diglu-conate, Aroma, Hydroxypropyl Methylcellulose, O-Cymen-5-Ol, Menthol, Neohesperidin Dihydrochalcone, Vanillin, Lactic Acid, CI 16255, Limonene.

The experimental patient examination times were (D0) initial examination, (D7) clinical examination at one week of treatment, (D14) clinical examination at two weeks of treatment, and (D28) clinical examination at three weeks of treatment.

- Sample

The main objective of this study is to analyze the efficacy of a new chlorhexidine compound compared with the 0.12% chlorhexidine compound during periodontal maintenance of a patient with periodontitis grade I or II after scaling and root planning treatment.

Inclusion criteria were as follows: 1) patients between 18 and 65 years of age, 2) patients with stage I or II periodontitis who are to receive periodontal treatment, 3) patients with an adequate level of understanding of the clinical research they are to participate in, who agree to follow the study procedures and provide an autonomous and signed informed consent to that effect, 4) patients without allergies, 4) patients with no allergies, hypersensitivity or any other type of incompatibility with any of the components of the investigational products, 5) patients with no diseases requiring therapies that interfere with the evaluation of the investigational products, 6) patients in good general health, 7) patients with at least 20 teeth present, and 8) patients with availability to attend the control appointments.

The exclusion criteria were: 1) patients taking antibiotics for any pa- topology or who had received antibiotics prophylactically one month before the start of the study, 2) pregnant patients, 3) patients with diabetes mellitus, and 4) patients taking medication related to gingival alterations. The participants were randomly assigned to each group using a numerical randomization list: group 1 (control) for treatment A (Chlorhexidine 0.12%) and group 2 (experimental) for treatment A (Chlorhexidine 0.12%).12%) and group 2 (experimental) for treatment B (new chlorhexidine mouthwash). The patients and the researcher providing the treatments and conducting the patient assessment were blinded. All study participants were given a bottle of mouthwash, a measuring cup, and a sheet with instructions for use. The application method was rinsing with 15ml of the solution for 1 minute every 12 hours for 28 days. Patients were told the importance of not applying other oral hygiene products while in the study and maintaining their usual oral hygiene habits. Patients were also asked to discontinue treatment and contact the research center if they experienced any adverse reactions.

All patients participating in the study were previously informed about the study's type and procedures, and they signed an informed consent form before the start of the study.

- Evaluation and follow-up visits

The trial includes four control times:

D0: Initial visit

D7: Visit seven days after initiation of treatment

D14: Visit 14 days after initiation of therapy.

D28: Visit 28 days after starting treatment. Final check-up.

At all control times, a clinical and periodontal inspection was carried out by means of a periodontal probing and periodontogram, and data referring to the variables to be analysed were collected.

Likewise, at the final visit (D28), the bottles were collected from all patients to evaluate each patient's compliance at the end of the study through the remaining mouthwash samples.

- Statistical Analysis

For the evaluation of the products, the following controls were carried out:

Main parameters

Plaque Index (PI): plaque level rating by percentage (0-100%).

Gingival Index (GI): classification of inflammation using a 4-point scale (0: no inflammation, 3: severe).

Bleeding index (BoP): percentage classification of bleeding intensity (0-100%).

Probing depth (PS): evaluation in millimeters.

Attachment Loss (CAL): assessment of attachment loss in millimeters.

Descriptive statistical analysis of quantitative variables: A descriptive statistical analysis of the quantitative variables at the different experimental times, including mean, standard deviation, and absolute variation concerning the baseline time and Treatment A, is carried out.

Normality of numerical variables: The Kolmogorov-Smirnov test has been applied to determine the normality of the variables. It is concluded that the variables under analysis do not have a normal distribution: IG_D0. IG_D7, IG_D14, IG_D28, IG_D7_0. IG_D14_0 AND IG_D28_0.

Cross-tabulation between qualitative variables: The Chi2 test was carried out.

To determine the groups that make the difference, we used Haberman's corrected standardized residuals, which allowed us to obtain the significance of the cells independently; this significance implies that the percentage of the cell is statistically different from that corresponding to the total of the sample.

Cross-tabulation of categorical variables and numerical variables: The ANOVA test has been applied.

Statistical significance: This has been indicated in the usual format (*p*<0.05; *p*<0.01; *p*<0.001, *p*<0.0001, and *p*<0.00001); the lower the Figure, the higher the significance.

## Results

The above experiments report auspicious results for combining chlorhexidine with cymenol due to its high penetrability (18). However, we know that the results of controlled *in vitro* studies may be difficult to extrapolate to an *in vivo* study without detracting from the fact that *in vitro* studies are a fundamental basis for further human development and research.

Reviewing the published literature, it is considered that the mouthwash references are adequate for a bioadhesive gel, as both presentations do not require rinsing after use, remaining in the mouth for some time, and it is essential not to ingest food or drink for 30 minutes after application of the product. This, together with the fact that from an experimental point of view, a mouthwash facilitates the application by the patient, eliminating the bias of possible errors in the administration of the product to be evaluated, the results of the comparison of the 0.12% chlorhexidine mouthwash vs. treatment with new chlorhexidine mouthwash composed of Aqua, Glycerin, Propylene Glycol, PEG-40 Hydrogenated Castor Oil, Xylitol, Sucralose, Chlorhexidine Digluconate, Aroma, Hydroxypropyl Methylcellulose, O-Cymen-5-Ol, Menthol, Neo-hesperidin Dihydrochalcone, Vanillin, Lactic Acid, CI 16255, Limonene.

- Number and characteristics of patients

Ninety-two patients were assessed in a first pre-screening visit at the dental clinic to determine whether they had the required degree of periodontitis. Of these, 43 patients were included in the study and underwent the corresponding scaling and root planing treatment. The final number of patients in the study was 40 (Table 1). Three patients (V19, V21, and V33) dropped out of the study for reasons unrelated to the study.

- Primary variables by treatment

See Table 2.

- Treatment-derived variables

See Table 3

## Discussion

This study's results show that chlorhexidine is indispuTable as an adjunctive method in the treatment of gingival and periodontal pathology, as widely supported by the literature.

Conversely, chlorhexidine produces long-term extrinsic staining and gingival in-flammation (19), which is why it is sought to compare its effects with those of other compounds used as an alternative or combined with chlorhexidine.

This study uses the combination with cymenol, which does not provide statistically significant results. Still, in recent years, compounds have been presented that could be a substitute for the "gold standard," which is 0.12% chlorhexidine, to avoid the adverse effects of the same and to be used by patients allergic to it, showing satisfactory results in terms of reduction of plaque index, gingival and bleeding index, probing depth and insertion loss (19).

Natural methods are an essential asset. The study published by Oak *et al*. in 2023 shows significant results regarding the use of different herbal mouth rinses, with HiOra (19) being the one with the best parameters, which is also reflected in the study by Matthew *et al*. (20), Hi-Ora shows a mean gingival index of 0.66 ± 0.16, and in the 0.12% chlorhexidine group, it is 0.70 ± 0.25. Similarly, regarding the plaque index in the chlorhexidine group, the results reported values of 0.80 ± 0.31, compared to 0.77 ± 0.30 in the Hi-Ora group. This product is an herbal compound containing Salvadora persica, Terminalia bellerica, Gandha purataila, and Piper betel and has been shown to have anti-plaque, antimicrobial, antiseptic, and analgesic effects (20,21).

Along the same lines, other studies, such as that of Penmetsa *et al*. (2020) (22), compare other natural compounds, such as Aloe Vera or Triphala-based compounds, with the latter showing better results in terms of plaque index, gingival inflammation, and bleeding level. Other natural methods, such as green tea rinses (0.5%) or aloe vera, show similar results to chlorhexidine 0.12-0.2% in treating gingivitis. Still, there are no specific case reports in stage I and II periodontal patients, as is the aim of this study (23).

A systematic review and meta-analysis published in 2021 by Al-Maweri *et al*. (24) reported no statistically significant difference in the reduction of dental plaque and gingival inflammation index between patients treated with chlorhexidine and those treated with turmeric rinses, which is supported by another systematic review of the same year (25) where the results are the same but stressing a low level of evidence. In other words, both types of mouthwash reported similar beneficial results at 3-, 4-, 6-, and 12-weeks follow-up.

In another line, probiotics are "live micro-organisms that, when provided in the right amounts, confer health to the individual" (26). Their ability to lower the pH of the oral cavity, their anticariogenic action, and their ability to facilitate the release of sub-stances that prevent the formation of bacterial plaque have been demonstrated (27). Henrique Soares *et al*., in their systematic review published in 2023 (28), reflect their role in improving periodontal health, comparing them with chlorhexidine, but did not obtain statistically significant results about the parameters gingival index and plaque index, which is supported by other studies (29). What is certain is that probiotics have been shown to help reduce the percentage of periodontopathogenic bacteria forming the "Red Complex" 6 months after essential periodontal treatment in patients with early-stage periodontitis (29).

Although 0.12% chlorhexidine is considered the "Gold Standard," the variation in the percentage of chlorhexidine and how it affects the results obtained has also been studied in the literature. Lee *et al*. (30) report better results in periodontal patients as the concentration of the compound increases. The bacterial network comprising the afore-mentioned "Red Complex," calculated by the BANA test, was reduced in the group treated with chlorhexidine gel to 0.44 ± 0.61 at eight weeks of follow-up after non-surgical treatment compared to the 0.12% chlorhexidine mouthwash, whose results at eight weeks of follow-up reported values of 1.05 ± 0.51 (*p*<0.0001). The same is true for the gingival index values, where the same study reveals optimal values for chlorhexidine at higher concentrations at 4- and 8-weeks follow-up (0.41 ± 0.34 vs. 0.65 ± 0.29; *p*<0.03).

A review of the literature on new alternatives to 0.12% chlorhexidine shows that no *in vivo* studies are focusing on the effect of cymenol as a bioadhesive agent, which is the focus of this study. Cymenol is a phenolic compound derived from isopropyl cresol (31). Although no statistically significant differences were found between the two treatments in the parameters evaluated, it is essential to note that the cymenol-based composite showed a promising trend in terms of biofilm removal in *in vitro* studies (18). This reinforces the need to consider new therapeutic options that may be viable alter-natives to the gold standard represented by 0.12% chlorhexidine, especially for patients with known allergies to this agent.

The null hypothesis that the newly designed compound of 0.12% chlorhexidine + 0.10% cymenol is not inferior in terms of efficacy when compared to 0.12% chlorhexidine is therefore accepted.

## Conclusions

In this comparative study between 0.12% chlorhexidine and 0.12% chlorhexidine with 0.10% cymenol as adjunctive therapy in the periodontal maintenance of patients with periodontitis grade I or II post scaling and root planing, no statistically significant differences were found in the clinical parameters evaluated, fulfilling the predefined criteria of non-inferiority. Although cymenol mouthwash showed promising results in biofilm removal *in vitro* studies, its clinical efficacy did not significantly outperform standard chlorhexidine.

This study highlights the importance of exploring new therapeutic alternatives that can balance the efficacy of chlorhexidine without the associated adverse effects, such as extrinsic staining and gingival inflammation. The search for bioadhesive agents such as cymenol could represent a promising direction for future research, especially in patients with sensitivity to chlorhexidine.

The findings underline the continued need for studies that evaluate the clinical effectiveness and the long-term tolerability of adjunctive periodontal treatments. Further research is required to fully elucidate the potential of cymenol and other compounds as viable alternatives to the current standard, aiming to improve quality of life and treatment adherence in patients with periodontal disease.

In conclusion, although this study did not demonstrate the superiority of the cymenol compound, it provides a solid basis for further exploration of options to optimize the balance between clinical efficacy and long-term tolerability in the treatment of periodontal disease.

## Figures and Tables

**Table 1:** Categorical identification variables.

Variable	Categories	Frequency	Percentage (%)
Treatment	Treatment A	19	47.5
	Treatment B	21	52.5
Sex	Woman	28	70.0
	Man	12	30.0
Age categorized	Up to 51 years old	19	47.5
	Over 51 years old	21	52.5

Treatment A: Chlorhexidine 0.12% mouthwash Treatment B: New Chlorhexidine 0.12% mouthwash + Cymenol 0.10%.

**Table 2:** Primary variables by treatment.

Variable	Treatment A		Treatment B		Significance		
	Mean	S.D.	Mean	S.D.	A<>B	A>B	B>A
Plaque Index (D0)	33.63	21.26	35.57	20.68	NS	NS	NS
Plaque Index (D7)	9.16	10.12	11.05	12.80	NS	NS	NS
Plaque Index (D14)	11.37	13.53	9.10	9.04	NS	NS	NS
Plaque Index (D28)	8.26	8.33	6.05	6.72	NS	NS	NS
Gingival Index (D0)	2.47	0.61	2.33	0.66	NS	NS	NS
Gingival Index (D7)	1.74	0.65	1.29	0.78	cuasi	*p*<0.05	NS
Gingival Index (D14)	1.37	0.68	1.00	0.84	NS	NS	NS
Gingival Index (D28)	1.00	0.88	0.71	0.64	NS	NS	NS
Gingival Index (D0)	34.79	20.92	32.76	18.90	NS	NS	NS
Bleeding Index (D7)	14.42	12.94	9.76	10.60	NS	NS	NS
Bleeding Index (D14)	13.32	12.97	10.00	13.08	NS	NS	NS
Bleeding Index (D28)	6.67	6.83	6.25	7.58	NS	NS	NS
Probing Depth (D0)	3.48	0.63	3.35	0.73	NS	NS	NS
Probing Depth (D7)	3.04	0.62	3.00	0.50	NS	NS	NS
Probing Depth (D14)	2.92	0.52	2.88	0.45	NS	NS	NS
Probing Depth (D28)	2.86	0.41	2.77	0.44	NS	NS	NS
Level of Attachment (D0)	-3.24	0.64	-3.07	0.76	NS	NS	NS
Level of Attachment (D7)	-2.88	0.60	-2.75	0.55	NS	NS	NS
Level of Attachment (D14)	-2.74	0.56	-2.62	0.50	NS	NS	NS
Level of Attachment (D28)	-2.74	0.43	-2.52	0.46	NS	NS	NS

**Table 3:** Treatment-derived variables.

Variable	Treatment A		Treatment B		Significance		
	Mean	S.D.	Mean	S.D.	A<>B	A>B	B>A
Variation of Plaque Index (D7-D0)	-24.47	20.78	-24.52	21.17	NS	NS	NS
Variation of Plaque Index (D14-D0)	-22.26	25.78	-26.48	20.60	NS	NS	NS
Variation of Plaque Index (D28-D0)	-25.37	25.00	-31.05	19.94	NS	NS	NS
Variation of Gingival Index (D7-D0)	-0.74	0.45	-1.05	1.02	NS	NS	NS
Variation of Gingival Index (D14-D0)	-1.11	0.88	-1.33	1.02	NS	NS	NS
Variation of Gingival Index (D28-D0)	-1.47	0.96	-1.62	0.86	NS	NS	NS
Variation of Bleeding Index (D7-D0)	-20.37	19.79	-23.00	21.19	NS	NS	NS
Variation of Bleeding Index (D14-D0)	-21.47	23.16	-22.76	13.39	NS	NS	NS
Variation of Bleeding Index (D28-D0)	-28.39	21.27	-26.90	15.22	NS	NS	NS
Variation of Probing Depth (D7-D0)	-0.44	0.68	-0.34	0.57	NS	NS	NS
Variation of Probing Depth (D14-D0)	-0.56	0.68	-0.47	0.56	NS	NS	NS
Variation of Probing Depth (D28-D0)	-0.56	0.70	-0.58	0.56	NS	NS	NS
Variation of Level of Attachment (D7-D0)	0.36	0.71	0.31	0.61	NS	NS	NS
Variation of Level of Attachment (D14-D0)	0.51	0.70	0.44	0.60	NS	NS	NS
Variation of Level of Attachment (D28-D0)	0.50	0.69	0.54	0.61	NS	NS	NS
